# Oxytocin and the Biopsychology of Performance in Team Sports

**DOI:** 10.1100/2012/567363

**Published:** 2012-09-10

**Authors:** Gert-Jan Pepping, Erik J. Timmermans

**Affiliations:** Center for Human Movement Sciences, University Medical Center Groningen, University of Groningen, 9713 AV Groningen, The Netherlands

## Abstract

Little is known about the biopsychological underpinnings of expert performance in team sports. In this paper we show that there is a vast support for oxytocin as a neuropeptide involved in the encouragement of important processes linked to greater team performance in sport. We argue that oxytocin is related to biopsychological processes aimed at convergence of emotions and moods between people, and in doing so it is a critical neuropeptide involved in the shaping of important team processes in sport such as trust, generosity, altruism, cohesion, cooperation, and social motivation, and also envy and gloating. Future research should examine the role of oxytocin in these essential components of sport performance. In particular, the link between oxytocin, emotional contagion and the cultivation of experiences of positive emotions is a worthwhile line of investigation for sport participation and development as well as high performance in sport.

## 1. Introduction

Prosocial behavior has been shown to serve an important purpose in enhancing future team performance in sport. For instance, Moll et al. [1] showed that in soccer penalty shootouts, players who engaged in certain prosocial celebratory behaviors were more likely to be in the team that ultimately won a penalty shootout. This was taken as evidence for the functional use of prosocial behavior such as expression and contagion of positive emotions in sport. Other psychological research has also related such behaviors to greater performance in various achievement settings (e.g., [2–10]), making them important to consider in elite sport performance. Neuroscientific research has shown that positive social interactions are linked to the neuropeptide oxytocin [11, 12]. The central question we address here is whether oxytocin may play a role in the biopsychological underpinnings of expert performance in team-sports. We will argue that since oxytocin is linked to key processes relevant to team sport, such as empathy, trust, generosity, altruism, cohesion, cooperation, and motivation, it provides an important biopsychological basis for expert performance in (team) sports. 

The experience and expression of social emotions are important aspects of human life. Emotions are meaningful in social contexts. They serve various communicative and motivational functions, which differ across personality, social, cultural, and situational variables [13, 14]. There are many suggested definitions of emotions [15], but central to its notion is the idea that components such as appraisals, experiences, expressive behaviors, and physiology interact over time to give rise to emotional states. These components interact not only within the individual but across people as well [16].

The expressions of emotions play an important role in the communication of social goals in sports in cooperative as well as competitive settings. Emotions have the capacity to communicate goals, needs, and intentions between people. Athletes freely express their emotions and often share them [17]. In the psychological and sport literature positive emotions have garnered relatively little empirical attention compared to negative emotions [2, 3]. However, research has shown that positive emotions have profound influences on a number of processes, including attentional control, cognition, and interpersonal functioning [2, 18–20]. Further, in a recent review McCarthy [3] linked subcomponents of sport performance, including social motivation, perception, attention, memory, decision-making and judgment, to positive emotions. Motivation and commitment are essential psychological attributes for successful performance in sport, and one factor to sustain motivated behavior and commitment is sport enjoyment [3]. Not only does enjoyment serve as motivational role in sport, but it is also supposed to be integral to optimal performance.

A neuropeptide that can be consistently linked to positive emotions as well as to the social brain is oxytocin [11]. Oxytocin is a neuropeptide of nine amino acids that is primarily synthesized in specialized cells in the paraventricular and supraoptic nuclei of the hypothalamus [21]. Oxytocin serves dual roles as both a central neurotransmitter/neuromodulator and a peripheral hormone. Peripherally, oxytocin regulates uterine contractions during labor and milk ejection during lactation. Centrally, oxytocin affects many brain regions, including the key emotional brain regions amygdala and the hypothalamus, where it acts as a neurotransmitter [22]. In humans as well as in other species, oxytocin is involved in the regulation of positive social behaviors and cognition, including partner preferences, approaching behavior, parental behavior, sexual behavior, social memory, pair bonding, social recognition, and social attachment behavior [11, 23–33]. In animals oxytocin and its role in the regulation of behavior are studied, for instance, by injecting synthetically produced forms of oxytocin in the brain. In humans most research either administers synthetic oxytocin using a nasal spray or monitors blood plasma levels following or during experimental manipulations.

There is very little research directly investigating oxytocin in sport. Existent literature centers on exercise physiological findings. Chicharro et al. [34] investigated the plasma oxytocin response to exercise in professional male cyclists by using a maximal incremental exercise test on a cycle ergometer. It was concluded that plasma oxytocin shows no response to graded exercise until exhaustion in professional cyclists. Hew-Butler and colleagues [35] examined acute changes in endocrine and fluid balance markers during high-intensity, steady state, and prolonged endurance running. They found unexpected increases in oxytocin. Hew-Butler et al. [35] suggested that oxytocin might play a role in the regulation of fluid balance during conditions of extreme physical stress.

In sum so far, emotions are present in all aspects of human life, including sport and exercise. In sport there are many social emotional interactions and emotions play a critical role in achieving successful sport performance. There is a need to increase our understanding of the social emotional foundations of sport behavior. To increase our understanding of the biopsychological underpinnings of performance it is important to bring together the fields of affective neuroscience, psychology, and neurophysiology in a sport context. To that end, in the following sections we will link oxytocin to psychological processes relevant to performance in a team-sport setting by discussing research on oxytocin and (i) social emotions such as empathy, trust, generosity, altruism, cooperation, and (social) motivation; (ii) perceptual processes such as emotion recognition of facial expressions and gaze behavior.

## 2. Oxytocin and Social Emotions

In sport, there are many social emotional interactions. Social emotions have been suggested to be causes for emotions in the people observing these emotions. The expression of an emotional state in one person leads to the experience of similar emotions in a person observing the expression. Through this process, emotions influence other's people's emotions, feelings, and behaviors, leading to the convergence of emotions and moods [14].

### 2.1. Empathy

Human *empathy* is a broad concept that refers to the cognitive as well as the emotional reactions of one individual to the observed experiences of another [36, 37]. Cognitive empathy is described as a cognitive role-taking ability, or the capacity to engage in the cognitive process of adopting another's psychological point of view [38]. The capacity to experience affective reactions to the observed experiences of others or share a “fellow feeling” has been described as emotional empathy and involves several underlying processes, including emotional contagion and shared pain [37]. So, on one hand, emotional contagion increases empathy. But on the other hand, the higher persons rate on scales measuring empathy, the more they engage in mimicry and the more susceptible they are to emotional contagion [39, 40]. Athletes often have to infer internal states of another person in order to make sense of or predict another person's behavior. This is a very important ability for athletes, because they need to know what actions or intentions are afforded by a teammate or an opponent. 

Hurlemann and colleagues [41] investigated whether oxytocin increased empathy measured by the Multifaceted Empathy Test (MET) [42], a test that provides behavioral indices of cognitive and emotional empathy. Findings revealed that emotional empathy was increased in response to both positive and negative valence stimuli. They also found that after intranasal oxytocin treatment, emotional empathy responses in men were raised to levels similar to those found in untreated women.

Barraza and Zak [43] investigated whether the experience of empathy raises oxytocin and demonstrated a positive relationship between the degree of empathy and the change in oxytocin plasma levels. Viewing an emotional video raised oxytocin plasma levels by an average of 47% over baseline compared to those who watched emotionally neutral video and after empathic responses were experienced. 

Domes et al. [44] hypothesized that oxytocin promotes the ability to infer the internal state of another person to adapt one's own behavior. Specifically, oxytocin was expected to improve performance on the Reading the Mind in the Eyes Test (RMET) [45], a task testing the ability to infer the affective mental state of others from subtle facial cues. Intranasal oxytocin improved performance on the RMET compared with placebo. Bartz et al. [46] also investigated the effects of oxytocin on cognitive empathy. In particular, they examined whether normal variance in social proficiency moderates the effects of oxytocin on social-cognitive performance. Social competencies of the participants were measured with the Autism Spectrum Quotient (AQ) [47]. Participants received intranasal oxytocin or a placebo and performed an empathic accuracy task measuring social-cognitive abilities. The findings showed that oxytocin improved cognitive empathy in less socially proficient individuals. 

### 2.2. Trust, Generosity, and Altruism

Sharing social emotions, by way of emotional contagion, play an important role in *trust, generosity, *and *altruism*. People who are mimicked and those who mimic another person both donate more money to a charity than controls [48, 49]. In addition, being mimicked appears to increase trust and generosity. These are important psychological processes in team sports because they enhance team cohesion, a dynamical process that is reflected in the tendency for a group to stick together and remain united in the pursuit of its instrumental objectives and satisfaction of member affective needs [50]. High team cohesion is associated with better sport performance [51, 52].

Kosfeld et al. [53] compared trusting behavior in a group of subjects that received a single dose of intranasal oxytocin with that of subjects in a control group that received placebo. They analyzed the effect of oxytocin on individuals' decisions in a trust game with real monetary stakes. Oxytocin caused a substantial increase in trust among humans. Zak et al. [54, 55] also found that oxytocin increased the feeling of trust in neuroeconomic experiments. Plasma oxytocin levels increased in response to a social signal of trust or in situations where there was a social intention of trust. In addition, oxytocin levels correlated with trustworthy behavior (the reciprocation of trust). Baumgartner and colleagues [56] found that participants that were administered the oxytocin did not show any change in their trusting behavior after they learned that their trust had been breached several times while subjects receiving placebo decreased their trust. Mikolajczak et al. [57] investigated whether the effects of oxytocin on human trusting behavior depended on context. In an economic trust game, Mikolajczak and colleagues [57] found that oxytocin enhanced trusting behavior, but that it facilitated such behavior only in the absence of cues that a social partner may be untrustworthy.

Zak et al. [58] concluded that oxytocin increases generosity in humans. They investigated, by manipulating oxytocin, the role of empathy in producing generosity. Zak et al. [58] tested whether oxytocin is a proximate mechanism prompting generosity between anonymous human strangers. Two tasks, the ultimatum game and the dictator game, were used to dissociate the physiologic role of empathy in producing generosity and altruism using monetary transfers. Participants were administered intranasal oxytocin or placebo and engaged in a blinded one-shot decision on how to split a sum of money with a stranger that could be rejected. Those on oxytocin were 80% more generous than those given a placebo. Oxytocin, however, had no effect on a unilateral monetary transfer task dissociating generosity from altruism. Barraza and Zak [43] also found that oxytocin modulated generosity in an economic game and charitable donations, a finding replicated by Barraza et al. [59]. In their study oxytocin did not significantly increase the decision to donate, but people infused with oxytocin were found to donate 48% more to charity than those given a placebo.

Kosfeld et al. [53] and Baumgartner et al. [56] demonstrated that people on oxytocin were more willing to entrust someone with their money than people on placebo. Their explanation for these results was that oxytocin increases trust and reduces the perceived risk of being betrayed. However, it is possible that participants made higher transfers not because they were more trusting but because they were simply being more generous [43, 58]. Mikolajczak et al. [60] aimed to rule out this alternative explanation and examined whether the trust enhancing effects of oxytocin could be extended to other, nonmonetary, scenarios. They used a paradigm in which subjects' trust behavior did not benefit the recipient (thereby controlling for the influence of generosity) and in which the variable at stake was not money but confidential information. Sixty healthy young adult men were randomly assigned to receive either intranasal oxytocin or placebo. Before substance administration, participants completed a questionnaire with very intimate questions. Participants were given an envelope for their completed questionnaire and the experimenter assured the participants that he would not look at their answers. However, the participants were free to seal the envelope. The degree of the envelope's opening (sealed plus taped, only sealed, or left open) was considered as a measure of the participant's trust in the experimenter. The results showed that 80% of the participants in the placebo group sealed the envelope and added tape, whereas only 7% in the oxytocin group did. Conversely, 60% of the participants in the oxytocin group left the envelope open, while 3% of the placebo group did. In addition, participants on oxytocin were 44 times more trusting that their privacy would not be violated than participants on placebo. Mikolajczak et al. [60] concluded that oxytocin does increase trust, and that its effects extend beyond money.

De Dreu et al. [61] investigated the effect of oxytocin on parochial altruism, that is, a preference for favoring the members of one's ethnic, racial, or language group. Their findings indicated that oxytocin promoted in-group trust and cooperation, and defensive, but not offensive, aggression toward competing out-groups. De Dreu et al. [62] examined the effect of oxytocin on human ethnocentrism, that is, the tendency to view one's group as centrally important and superior to other groups. It has been suggested that ethnocentrism facilitates within-group trust, cooperation, and coordination. It was found that oxytocin creates intergroup bias and motivates in-group favoritism and, to a lesser extent, out-group derogation. De Dreu et al. [62] concluded that oxytocin promotes human ethnocentrism.

### 2.3. Envy and Gloating

If an interaction is antagonistic rather than affiliative it may be that contrasting emotions occur in two parties not because nonverbal actions are matched but because they tend to elicit oppositional kinds of response in the other [14]. Such situations are common in sports, in which competition and opposing goals are important. Two important social emotions which often occur in a competitive sport context are *envy* and *gloating*. Shamay-Tsoory et al. [63] examined the effect of oxytocin on envy and gloating. Envy is defined as a negative emotional reaction in the face of another person's good fortune [64] and gloating is the malicious pleasure at the other's misfortune [65]. Shamay-Tsoory and her colleagues [63] used a paradigm of a game of chance involving monetary gains, which was designed to elicit these emotions in a highly controlled setting. Following the intranasal administration of oxytocin or a placebo, participants played a game of chance with another (fake) participant who either won more money (envy manipulation), lost more money (gloating manipulation), or won/lost equal amounts of money. Their results showed that in comparison with the placebo, oxytocin increased the envy ratings during unequal monetary gain conditions involving relative loss (when the participant gained less money than another player). Oxytocin also increased the ratings of gloating during relative gain conditions (when the participant gained more money than the other player). By contrast, oxytocin had no effect on the emotional ratings following equal monetary gains. Thus, their results suggested that the oxytocinergic system is involved in modulating the negative social emotions envy and gloating. This is in contrast with the prevailing belief that the oxytocinergic system is specifically involved in positive prosocial behaviors. 

### 2.4. Cooperation and Motivation

It has been shown that the contagion of positive emotions between group members improves *cooperation *[66]. Mutual cooperation is an important component of team sport performance. Mutual cooperation requires the simultaneous fulfillment of being willing (*motivated*) to cooperate and trusting that the other(s) will cooperate as well. To get more insight in the role of oxytocin in cooperative behavior, Declerck et al. [67] examined the impact of oxytocin, incentives, and social information on cooperative decision-making in interdependent and uncertain social exchanges. They hypothesized that oxytocin positively affects cooperation only when social information is present, and that this effect is more apparent in situations with strong cooperative incentives than in situation with conflicting cooperative motives. Participants received intranasal oxytocin or a placebo and played two economic games: a coordination game (with strong incentives to cooperate) and a prisoner's dilemma game (with weak cooperative incentives). The presence or absence of social information was manipulated in the form of prior contact. The results showed that oxytocin enhanced cooperation only when social information was present, and this effect was significantly more pronounced when strong cooperative incentives were presented. When no prior social contact was made, oxytocin decreased cooperation. 

Rilling et al. [68] investigated the impact of intranasally administered oxytocin and vasopressin on cooperative behavior and brain activity among men in the context of an iterated prisoner's dilemma game. Vasopressin, an anxiogenic neuropeptide, is thought to play a role in intermale aggressive communication [69, 70]. As opposed to oxytocin, Rilling et al. [68] hypothesized vasopressin to be associated with decreased rates of cooperation in male subjects. The results showed that oxytocin, relative to both vasopressin and placebo, increases the caudate nucleus response to reciprocated cooperation, which may increase the reward of reciprocated cooperation and/or facilitate learning that another person can be trusted. In addition, Rilling et al. [68] found that oxytocin was associated with increased rates of cooperation following unreciprocated cooperation in the previous round compared with vasopressin.

Alvares and colleagues [71] investigated whether oxytocin ameliorated the acute behavioral and affective consequences of social rejection by using a virtual ball tossing game. Healthy male and female participants were administered intranasal oxytocin or placebo and subsequently ostracized or included during this game. Outcomes indicated that ostracized participants, compared to those included, displayed stronger motivation for inclusion and affiliation with other participants. These effects were not influenced by oxytocin. Intranasal oxytocin did, however, increase the desire to play again with the same participants, suggesting that oxytocin enhanced desire to future social engagement following inclusion.

In a recent review, Gordon et al. [72] examined the role of oxytocin as it relates to social motivation. Social motivation is the basic human need to become a member of groups organized around one's familial, cultural, religious, national, community, political, occupational, scholastic, and/or recreational identity [72]. By reviewing biological determinants of motivation in a social context during the course of development as well as genetic and epigenetic influences, they concluded that the oxytocin system is a key element in social motivation. 

In sum so far, the literature provides supportive evidence that oxytocin is linked to social emotions. Several studies about the role of oxytocin in social behavior have shown that it increases positive social emotions. Oxytocin is involved in empathy, trust, generosity, altruism, cohesion, cooperation and (social) motivation. The finding that oxytocin increases ratings of envy and gloating is important and has to be considered in the light of the competitive context in the Shamay-Tsoory et al. study [63, 73]. Envy is a consequence of the contagion of positive emotions in a competitive context, which is very common in a sport setting with two competing individuals or teams (e.g., in tennis and football). 

## 3. Oxytocin and Perceptual Processes

In (team) sports, the ability to recognize emotions accurately is important, because it is essential for the communication of social goals and intentions between team members. In addition, successful performance in a sport context requires skill in perception. For example, gaze is important to detect opportunities of actions for an athlete in a sport setting, but gaze also affects impressions and expectancies in sport. For example, Greenlees et al. [74] examined the impact of soccer players' gaze behavior on the impressions that are formed of them by opposing goalkeepers. Penalty takers displayed a different combination of gaze (either 90% or 10% with gaze operationalized as looking directly at the goalkeeper). The results revealed that those penalty takers displaying 90% gaze were perceived to possess positive characteristics to a greater extent than penalty takers displaying 10% gaze.

### 3.1. (Neural Responses to) Perception and Recognition of Emotions

Numerous studies have examined the role of oxytocin in the neural responses to emotions [86–89]. The overall conclusion of these studies is that oxytocin has modulatory effects on neural activity to facial emotional expressions. For example, Domes et al. [87] investigated the effect of intranasally administered oxytocin on brain activity in response to social emotional stimuli of varying valence in women. In a functional magnetic resonance imaging study women were presented with fearful, angry, happy, and neutral facial expressions after a single dose of intranasal oxytocin or a placebo administration in a within-subject design where no specific task was used. Group analysis revealed that the blood-oxygen-level-dependent signal was enhanced in the left amygdala, the fusiform gyrus, and the superior temporal gyrus in response to fearful faces and in the inferior frontal gyrus in response to angry and happy faces following oxytocin treatment. These areas have been implicated in the processing of facial emotions, gaze behavior and contain mirror neurons [91]. 

Marsh et al. [75] examined whether oxytocin enhances the recognition of positive facial affect. Volunteers were randomly assigned to receive intranasal oxytocin or placebo and accomplished a facial expression recognition task, which featured six basic emotions. Oxytocin increased sensitivity to positive emotional expressions. Participants who received oxytocin identified happy expressions more accurately than those who received placebo, whilst no significant differences in recognition of other expressions were found. Several other studies have also examined the modulatory effects of oxytocin on the recognition of facial expressions. Guastella et al. [76] found that oxytocin improved face identity recognition, particularly for faces displaying happy expressions. Di Simplicio et al. [77] found changes in response biases to neutral and surprise expressions, but no indications of superior processing of happy expressions. More recently, Schulze et al. [78] investigated the modulatory effects of oxytocin on the recognition of facial expressions under conditions of limited awareness. A single dose of intranasal oxytocin increased detection accuracy for very briefly (18, 35, or 53 milliseconds) presented emotional faces. This effect was more pronounced for the recognition of happy faces. 

Guastella et al. [79] examined the effect of oxytocin on the recognition of separate basic emotions with the aid of a dynamic facial expression recognition task. The task involved the use of computer-manipulated images of faces, whose neutral expression was gradually and continuously changing into an emotional one. A single dose of intranasal oxytocin or a placebo was administered to healthy male participants 45 minutes prior to task performance. The participants were instructed to press a stop button once they recognized an emotion emerging from the neutral face. A single intranasal administration of oxytocin improved the ability to recognize fear, but not other emotions. Lischke and colleagues [82] also focused on the effects of oxytocin on emotion recognition from dynamic facial expressions. It was found that oxytocin promoted emotion recognition from dynamical facial expression, as the oxytocin group recognized emotional expressions at lower intensity levels.

Guastella et al. [79] investigated the effect of oxytocin administration on emotion recognition of young people with autism spectrum disorders. They administered intranasal oxytocin or placebo to young male participants diagnosed with Autistic or Asperger's Disorder. After administration, all participants completed the RMET. The findings showed that in comparison with placebo, oxytocin administration improved performance on this task. This effect was also shown when analysis was restricted to the participants who received a lower dose. Guastella and his colleagues [79] concluded that oxytocin nasal spray improves emotion recognition for young people with autism spectrum disorders. In a recent study, Averbeck et al. [80] showed that intranasal oxytocin also improved the ability of patients with schizophrenia to recognize emotions.

### 3.2. Gaze Behavior and Other Perceptual Processes

Several studies have indicated that oxytocin influences gaze behavior of people. Guastella et al. [83] tracked the eye movements of healthy male volunteers presented with 24 neutral human faces after intranasal administration of oxytocin or placebo. Men administered with a single dose of intranasal oxytocin gazed longer and fixated more frequently toward the regions of neutral human faces critical for interpersonal communication. Oxytocin significantly increased gaze duration and fixation counts toward the eye region of human faces. These findings suggested that eye-gaze enhancement could be one of the mechanisms by which oxytocin enhances emotion recognition, interpersonal communication, and social approach behavior in humans. 

Guastella et al. [84] investigated whether oxytocin modified responses to positive and threatening social stimuli at an early perceptual stage of processing using a visual search task. Oxytocin or a placebo was administered to healthy volunteers who completed a visual search paradigm. The results demonstrated that angry faces were detected more efficiently than happy faces. In comparison to a discrepant happy face, participants were faster and more accurate when detecting an angry face in a crowd of both happy and neutral faces. Participants also gazed longer and more frequently toward angry faces. Oxytocin did not, however, influence response time, accuracy, or gaze toward angry or happy faces, even when participants were separated into high and low social anxiety. The findings from Guastella et al. [84] suggested that oxytocin may not influence the detection of positive and threatening social stimuli at early perceptual levels of processing.

Gamer et al. [85] investigated the neural basis of the modulatory effects of oxytocin on processing positive social stimuli and gaze-orientating behavior. Oxytocin had differential effects on the activity of specific amygdala subregions. It attenuated activation in lateral and dorsal regions of the anterior amygdala for fearful faces and enhanced activity for happy expressions. This indicated a shift of the processing focus toward positive social stimuli. Furthermore, oxytocin increased the likelihood of reflexive gaze shifts toward the eye region irrespective of the depicted emotional expression. 

Kéri and Benedek [90] investigated the effect of oxytocin on the perception of biological motion (a walking person) and nonbiological motion (a rotating shape). Healthy volunteers observed moving dots embedded among a cloud of noise (mask) dots. The task was to judge whether the target stimulus was present within the mask dots or not by pressing one of two different keys on the computer keyboard. The results showed that intranasal oxytocin administration, relative to placebo administration, increased sensitivity to biological motion but not to nonbiological motion.

In sum, numerous studies have shown that oxytocin enhances emotion recognition and modifies gaze behavior and other perceptual processes. Emotion recognition plays an important role in peoples' ability to experience affective reactions to the observed experiences of others [37]. Oxytocin does not seem to specifically improve emotion recognition of positive expressions. Various studies concluded that oxytocin administration improves the ability to recognize negative facial expressions as well. Overall, however, the literature showed evidence that oxytocin facilitates emotion recognition. Additionally, oxytocin seems to enhance gaze toward the eye region of faces. The eyes represent the communication focal points of the face and are the primary source for detection of interpersonal trust, threat, and emotions in others [92, 93]. Furthermore, oxytocin enhances the perception of biological motion in humans. The recognition of movements and actions of other people is necessary for communicating, decoding body language, and learning by imitation [94–96]. So, oxytocin increases gaze to socially meaningful information. This facilitates the perception and recognition of emotions in others, which plays an important role in for instance emotional contagion and the reading of socially relevant body language. 

Table 1 summarizes the evidence in relation to our hypothesis that oxytocin is linked to psychological processes relevant to team-sport performance.

## 4. Discussion 

**Figure 1:**
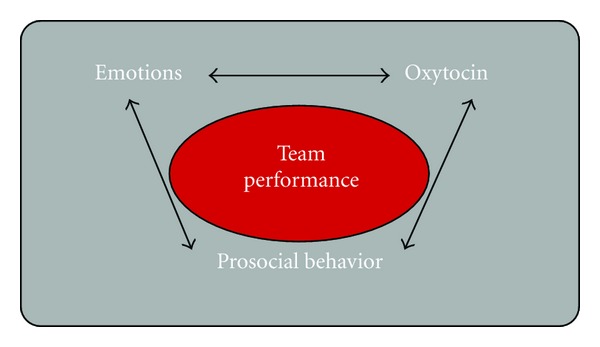
The association between social emotions, oxytocin, and prosocial behavior.

**Figure 2:**
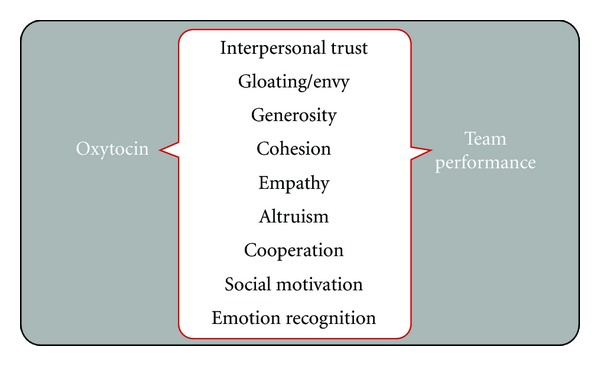
Oxytocin encourages important team processes.

An overview of research on oxytocin was presented with an aim to examine evidence on the link between oxytocin and psychological processes relevant to performance in a team-sport setting. Results of the review reveal an intimate link between oxytocin, social emotions, and prosocial behavior (see Figure 1). An important requirement in team sport is for athletes to work together. That is, in team sports individual players can inspire a team to perform better, whilst the team can inspire an individual player to optimally perform. Processes that play an important role here are, amongst others, (social) motivation, trust, emotional empathy, being able to read body language (mind reading), emotion recognition, generosity, and altruism. The results of our paper show oxytocin is importantly implicated in all of these processes (see Figure 2).

The ability to perceive and understand the mental state of others is very important for an athlete in a sport and especially so in a team setting where working together toward common goals is key. Athletes have to infer internal states in order to make sense of or predict a teammate or opponent's behavior. Mind, and its whole body equivalent reading, provides the athlete with information about what actions another person can perform and what actions another person affords the athlete [97, 98]. The current results suggest that oxytocin plays an important role in the psychological skills needed to enhance team performance. For instance, the findings of Moll et al. [1] discussed in the introduction, that prosocial celebratory behaviors were associated with enhanced team performance in the penalty shootout could be interpreted in terms of increased oxytocin levels and its behavioral effects.

All studies examining the effects of oxytocin on emotion recognition used facial expressions as stimuli. However, it has been shown that bodily expressions are recognized as reliably as facial expressions. De Gelder [99] reviewed a series of arguments in favor of substantially extending and enriching current human emotions theories by adding investigations of bodily expressions. For example, one important benefit to be gained from using bodily expression stimuli is the broader emotion perspective obtained by using affective signals that can operate over longer distances than faces [99]. In a sport setting, athletes need to be able to recognize emotional body expressions, because they need to communicate across great distances. Furthermore, focusing on facial expressions tends to make us refer to a person's mental state whilst focusing on bodily expressions directs attention to a person's or a group's actions [99], which is of course very important for athletes, in particular in team-sport situations. Further, research has shown that the display of body language affects how athletes perceived the opponent's confidence, competiveness, and focus. For example, Greenlees et al. [100] found that table-tennis players reported lower confidence in their ability to defeat the opponent when they viewed opponents wearing table-tennis specific clothing and opponent portraying positive body language than when they viewed opponents with negative body language or wearing general sportswear.

The current paper has shown that a lot of the research on oxytocin and social behavior is performed in abstract and artificially constructed contexts and the context of neuroeconomics and economic decision-making. It is recommended to study oxytocin and its links to social psychological processes in a sport context. Because of the natural availability and necessity of the processes involved, sport offers a very ecologically valid context to examine the effects of oxytocin. 

In this paper we hypothesized that oxytocin is linked to a range of psychological processes. However, at this stage it is important to acknowledge some complexities with regard to overly simplistic interpretations of the findings presented. For instance, although results show that oxytocin increases people's abilities to share positive emotions, which could be presumed to be good if it considers team members, results also show that under certain circumstances oxytocin promotes antisocial and negative emotions. The finding that oxytocin promotes ethnocentricity [62], in-group trust and cooperation and defensive aggression toward out-group [61], and envy and gloating [63] highlights the diverse effects oxytocin may have in team-sport settings. An orientation toward the own group and team can be a good thing in team-sport and used to enhance social and task cohesion [50]. However, envy and antisocial behaviors such as gloating and aggression can be very destructive in a sport context. A similar argument could be made for the role of empathy in a team-sport setting. Where increased empathy for a teammate is probably beneficial, it is not known whether empathy for an opponent or rival is useful in a performance environment and whether oxytocin increases or decreases empathy for an opponent. In the context of emotional contagion the question remains whether athletes are more likely to catch the emotions of teammates and those people that are liked than those that are disliked and hated or of those in the in-group/own team versus the out-group/opponents. It is important that further research addresses these issues in sport.

In conclusion, to gain a greater understanding of the psychology of team-sport performance and its biobehavioral underpinnings the fields of affective neuroscience, psychology and neurophysiology were brought together. In particular, we hypothesized a link between the neuropeptide oxytocin and important psychological team-sport processes. An overview of research was presented showing supporting evidence indicating a role of oxytocin in the biopsychology of team sport performance. Oxytocin has effects on cognitive empathy, emotional empathy, mind reading, positive and negative social emotions, emotion recognition, gaze behavior and the mirror neuron system may cause convergence of positive emotions and moods between people and make it possible that athletes can respond to the emotional behavior from their fellow players and opponents. So, there is considerable support for the hypothesis that oxytocin plays a role in enhancing team sport performance. In future, more research is needed to further investigate this theory. We predict that insight into the biobehavioral underpinnings of socioemotional processes will have significant importance for sport psychological and sport science support, talent identification, coaching, training, team selection, and team development.

## Figures and Tables

**Table 1:** Overview of studies reviewed (*n* = 36) that show supportive evidence for oxytocin as a neuropeptide involved in the encouragement of biopsychological processes linked to greater team performance in sport.

Biopsychological processes	Authors
Social emotions	
Empathy	[41, 43, 44, 46]
Trust	[53–57, 60–62]
Generosity	[43, 58, 59]
Altruism	[61]
Envy/gloating	[63]
Cooperation	[61, 62, 67, 68]
(Social) motivation	[61, 62, 71, 72]
Social perception	
Emotion recognition	[75–82]
Gaze behavior	[83–85]
Perception	[85–90]
